# Stability of nanofluids in quiescent and shear flow fields

**DOI:** 10.1186/1556-276X-6-231

**Published:** 2011-03-16

**Authors:** Sanjeeva Witharana, Haisheng Chen, Yulong Ding

**Affiliations:** 1 Institute of Particle Science and Engineering, University of Leeds, Leeds LS2 9JT, UK; 2 Institute of Engineering Thermophysics, Chinese Academy of Sciences, Beijing 100190, China

## Abstract

An experimental study was conducted to investigate the structural stability of ethylene glycol-based titanium dioxide nanoparticle suspensions (nanofluids) prepared by two-step method. The effects of particle concentration, fluid temperature, shear rate and shear duration were examined. Particle size and thermal conductivity measurements in quiescent state indicated the existence of aggregates and that they were stable in temperatures up to 60°C. Shear stability tests suggested that the structure of nanoparticle aggregates was stable in a shear interval of 500-3000 s^-1 ^measured over a temperature range of 20-60°C. These findings show directions to resolve controversies surrounding the underlying mechanisms of thermal conduction and convective heat transfer of nanofluids.

## Introduction

Nanofluids are suspensions of nano-sized particles in liquids, where particle sizes are preferably below 100 nm. At modest particle concentrations, the thermal conductivity, forced convective heat transfer, and critical heat flux of nanofluids were reported to be superior to respective base liquids [1-8]. In the backdrop of conventional heat transfer technologies approaching their upper limits, nanofluids are seen as a potential contender for small- and large-scale thermal applications [9-12]. A number of attempts had been made in the past, and postulates were put forward to explain the underlying mechanisms. Although yet inconclusive, the nanoparticle aggregation in liquids is believed to be one of the principal mechanisms behind the enhanced thermal conductivity and convective heat transfer [13-16]. In either case, the importance of particle aggregation and their stability were underlined.

On the other hand, the aggregation of nanoparticles is found to be the key mechanism behind the increase of nanofluid viscosity and shear thinning behaviour [14,17,18]. Recently, it was shown that the high shear viscosity of nanofluids could accurately be predicted by combining the conventional Krieger and Dougherty model and aggregation effects [18-20]. Those postulates were based on the assumption that, in the shear flow field, the aggregates will be stable because the hydrodynamic forces are insufficient to break the aggregates down to primary particles. However, the experimental evidences are insufficient to showcase the stability and particle structuring of nanofluids in flow conditions.

In the present study, the ethylene glycol (EG)-based Titania (TiO_2_) suspensions are selected to investigate the stability of nanofluids in quiescent and shear flow fields. Also their thermal conductivities are measured at various temperatures and compared with theoretical predictions. The experimental conditions were chosen resembling the possible industrial applications for nanofluids. Considering the bounded yet deep focus of the stability of nanofluids under different conditions, this article is reported as a letter without comparing the data with the other literature.

## Experimental

**Figure 1:**
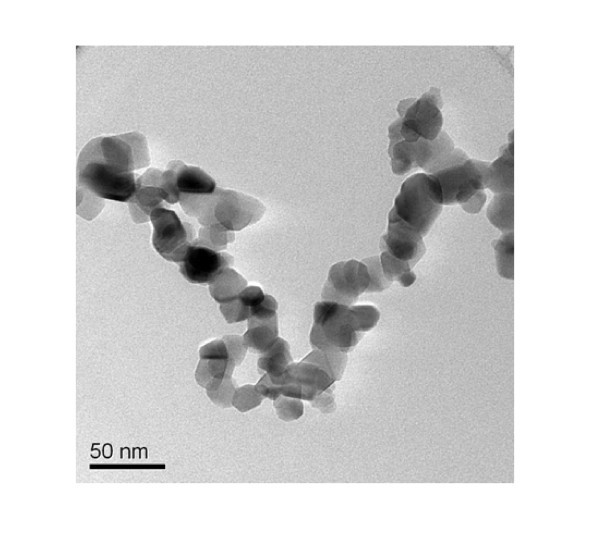
**Titania particles as received**.

Nanofluids were formulated using TiO_2 _nanopowder and EG. The dry TiO_2 _nanopowder purchased from Degussa Corporation in Germany was claimed to be consisting of spherical particles of 25-nm diameter. Electron microscopy (EM) imaging such as in Figure 1 suggests that the particles were in the form of agglomerates. In order to manufacture a stable nanoparticle suspension, a sequence of processes were followed. Further details of formulation can be found elsewhere [7,21]. The EM images of the nanofluid confirmed that the nanoparticles were well dispersed. Moreover, the light-scattering data collected using the Malvern Zetasizer-nano device showed that the suspended particles were in the order of around 130 nm in size. This is an indication of the formulation technique substantially reducing the aggregate size but failing to break them down to primary particles. This observation agrees with the recently concluded International Nanofluids Property Benchmarking Exercise (INPBE) [22]. These nanofluids were stable for 2 months without a visible separation, indicating the stability of aggregates in the long run.

Measurements of thermal conductivity (*k*, W/mK) of TiO_2_-EG nanofluids were conducted using the state-of-the art Lambda meter device acquired from PSL Measurement Systems GMBH of Germany. This instrument works on transient hot wire principle. For calibration with EG, the instrument reproduced the data up to 99% precision.

Shear flow field was applied to the samples using a Bohlin rotational rheometer. The experimental conditions were as follows: shear rates 500, 1000, 2000 and 3000 s^-1^; time durations 5, 10, 20 and 40 min; and temperatures 20, 30, 40, 50 and 60°C. These temperature and flow parameters were so chosen to suit possible industrial applications [19]. The shearing was preceded and followed by particle size measurements using Malvern Zetasizer-nano. The size measurements were repeated six times, and the reproducibility of data fell within error of 4%. In all instruments, the thermal equilibrium was ensured by leaving the samples at measuring temperature for a sufficient period of time before taking the readings.

## Results and discussion

**Figure 2:**
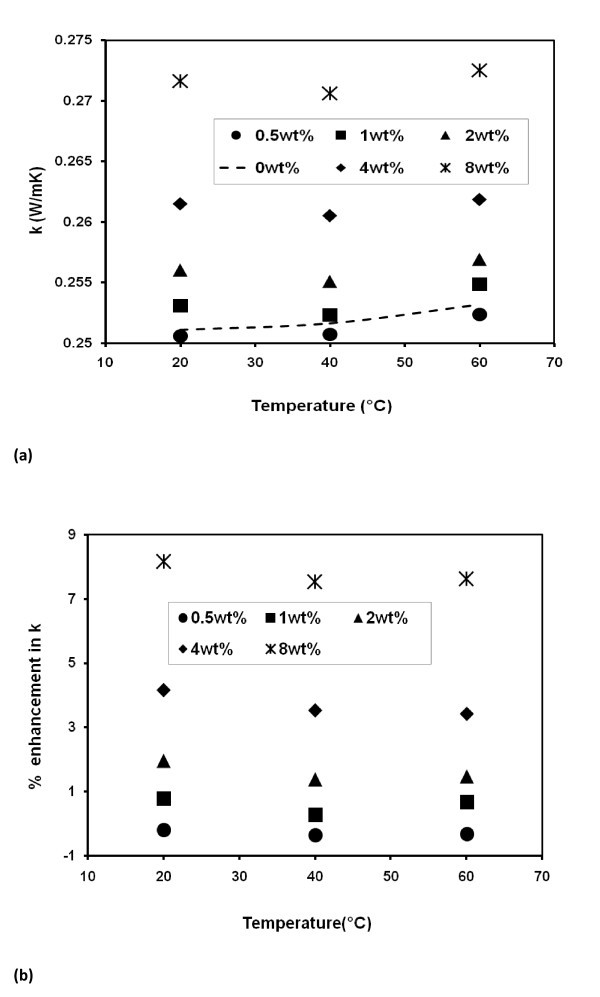
**Thermal conductivity of TiO**_**2**_**-EG nanofluids**.

Thermal conductivity (*k*, W/mK) data for the samples are presented in Figure 2. The trends of *k *of the nanofluid and base liquid appear alike. This follows that the presence of nanoparticles at these concentrations has not altered the dynamics of the base liquid. Interestingly, this was the case even at 60°C, indicating quiescent flow fields. Also shown in Figure 2 are the percentage (%) enhancements of thermal conductivity. At any given temperature, the enhancement has systematically increased with loading. However for a given concentration, the enhancement appears to be fairly stable with temperature. This is a trend that agrees with the more recent literature on this area [23,24]. Also noted from Figure 2 are the low particle loadings unable to cause noticeable enhancement. This observation contradicts a section of the old literature, while agreeing with majority of recent study including INPBE [22] participated by dozens of nanofluids research institutions.

**Figure 3:**
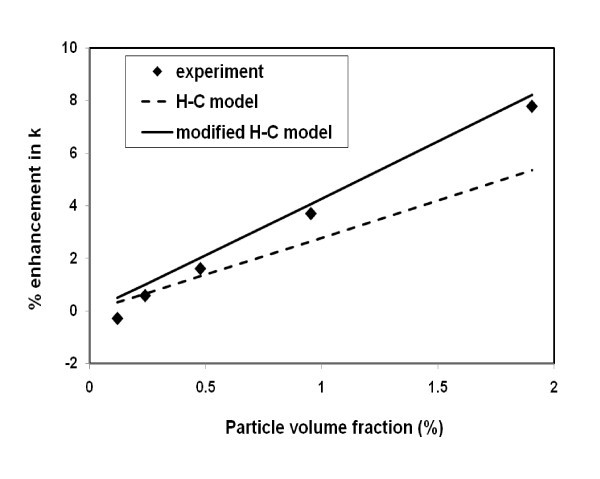
**Measured and predicted thermal conductivity**.

The average enhancement for each concentration in Figure 2b is plotted in Figure 3 together with the predictions of classical Hamilton-Crosser (H-C) model based on well-dispersed particles [25] and modified H-C model [20] based on aggregated particles. The classical H-C model can be written as(1)

where *k*, *k*_0_, *k*_p _are, respectively, the thermal conductivities of the nanofluid, base liquid, and particle material, and *n *is the shape factor given by *n *= 3/*ψ *with *ψ *the surface area-based sphericity (*ψ *= 1.0 for spheres).

Modified H-C model based on aggregated particles takes the form of [20](2)

where *k*_a _is the thermal conductivity of aggregates which is estimated by the Bruggeman model for spherical particles [26]:(3)

Here, *φ*_a _is the effective particle volume fraction given by φ_a _= φ(*a*_a_/*a*)^3-^^*D *^according with the fractal theory, and *φ*_in _is the solid volume fraction of aggregates given by φ_in _= (*a*_a_/*a*)^*D*^^-3^. Also *a *and *a*_a _are the radii of primary nanoparticles and aggregates, respectively [27], and *D *is the fractal index having a typical value of 1.8 for nanofluids [20]. From Figure 3, the conventional H-C model underpredicts the measurements by a considerable margin can be seen. However, the modified H-C model that takes into account the aggregates of nanoparticles agreed well with the experimental data.

Overall view of Figures 2 and 3 suggests that (i) the aggregation of nanoparticles is a principal mechanism that drives the thermal conductivity enhancement and (ii) the aggregates are stable in quiescent flow fields even at temperature as high as 60°C. Independence of the experimental data on temperature further suggests the weak or negligible effect of particle Brownian motion on reported enhancement.

**Figure 4:**
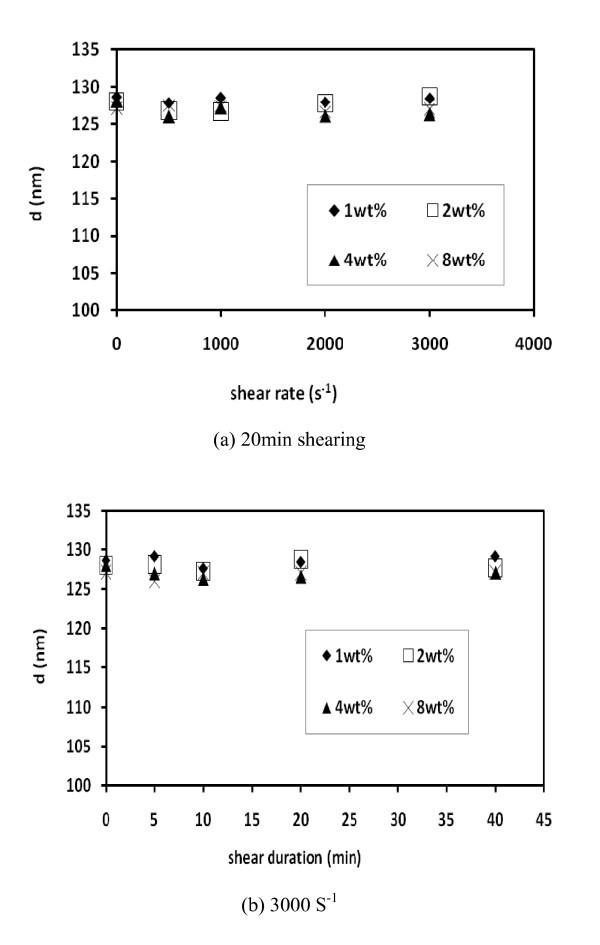
**Average particle sizes measured at 20°C**.

**Figure 5:**
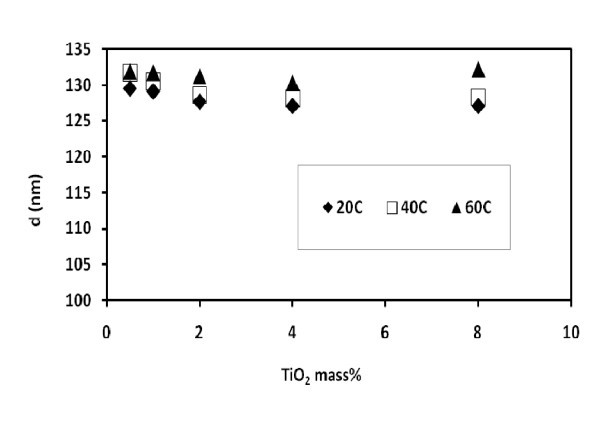
**Average particle size after 40 min of shearing at 3000 s**^**-1**^.

Featured in Figures 4 and 5 are the studies on particle size in shear flow fields. All samples have the measured particle sizes considerably larger than the primary size (25 nm) reconfirming the existence of the aggregates. Yet, the average particle diameter (*d*) exhibits a narrow fluctuation between 126 and 132 nm, which falls within the boundaries of experimental error. Moreover, the shear rates and shear durations shown on Figure 4 had been unable to break the aggregates. The aggregates were therefore sufficiently stable under these conditions.

Figure 5 illustrates the dependence of the measured particle sizes on the measuring temperature and particle concentration. At any given concentration, a temperature increase of threefold (from 20 to 60°C) has not registered a notable size change. Here, the indication is the temperature stability of aggregates. Furthermore, a concentration increase by 16 folds (from 0.5 to 8 wt%) has caused only a modest increase in size which again falls within the experimental error.

## Conclusions

Experiments were conducted to study the dependence of shear stability of nanofluids on temperature, particle loading and shear rate. Observed weak dependence of thermal conductivity enhancement on temperature supports the claim of particle aggregation as a principal mechanism behind the enhancement. Moreover, the aggregates in quiescent flow fields were stable in temperatures up to 60°C. The data on shear stability show that the aggregates are sufficiently stable over a range of rigorous shear rates and temperatures. The observations of thermal conductivity and particle size complement each other in terms of predicting the former from the latter. A comparison of the present findings with the literature data is currently underway and will be reported in future.

## Abbreviations

EG: ethylene glycol; EM: electron microscopy; H-C: Hamilton-Crosser; INPBE: International Nanofluids Property Benchmarking Exercise.

## Competing interests

The authors declare that they have no competing interests.

## Authors' contributions

The work presented here was carried out in collaboration between all authors. SW defined the research theme, designed methods, carried out the laboratory experiments, analysed the data, interpreted the results and wrote the paper. HC prepared the samples, helped to carry out the laboratory experiments, analysed the data and helped on writing the paper. YD defined the research theme and analyzed the data, All authors read and approved the final manuscript.
